# Activity of antifungal drugs and Brazilian red and green propolis extracted with different methodologies against oral isolates of *Candida* spp.

**DOI:** 10.1186/s12906-021-03445-5

**Published:** 2021-11-24

**Authors:** Ana Rita Sokolonski, Maísa Santos Fonseca, Bruna Aparecida Souza Machado, Kathleen Ramos Deegan, Roberto Paulo Correia Araújo, Marcelo Andrés Umsza-Guez, Roberto Meyer, Ricardo W. Portela

**Affiliations:** 1 Programa de Pós-graduação em Processos Interativos de Órgãos e Sistemas. Instituto de Ciências da Saúde, Universidade Federal da Bahia, Salvador, BA 40110-100 Brazil; 2 Instituto de Tecnologias de Saúde, SENAI/CIMATEC, Salvador, BA 41650-010 Brazil; 3 Departamento de Biotecnologia, Instituto de Ciências da Saúde, Universidade Federal da Bahia, Avenida Reitor Miguel Calmon s/n, Vale do Canela, Salvador, Bahia State 40110-100 Brazil

**Keywords:** Antifungal activity, Fungal biofilm, Green propolis, Oral candidiasis, Red propolis

## Abstract

**Background:**

Oral candidiasis is an opportunistic disease caused by fungi of the *Candida* genus. The occurrence of *Candida* spp. resistance to the commercial antifungal drugs points to the search for alternative treatments. Propolis has been successfully used in the treatment of infectious diseases for centuries. It has been proposed that an ultrasound pretreatment in the propolis extraction protocol can enhance the concentrations of molecules with antimicrobial activities in the final extract. Thus, this study aimed to compare the antifungal activity against oral *Candida* spp. isolates of green and red propolis extracts submitted or not to an ultrasound pretreatment before the extraction procedure.

**Methods:**

*Candida* spp. were isolated from denture stomatitis lesions and identified by sequencing. Oral *Candida* spp. isolates and reference strains were submitted to broth microdilution assays using commercial antifungals and Brazilian green and red propolis extracts submitted or not to an ultrasound pretreatment. Minimal Inhibitory Concentrations (MIC) and Minimal Fungicide Concentrations (MFC) were determined and biofilm formation interference was evaluated for resistant isolates.

**Results:**

*C. albicans*, *Candida tropicalis* and *Candida dubliniensis* were isolated from denture stomatitis lesions. Growth inhibition was observed in all *Candida* isolates incubated with all green and red propolis extracts. At lower doses, red propolis extracts presented significant antifungal activity. The ultrasound pretreatment did not promote an increase in the antifungal activity of green or red propolis. Three isolates, which were highly resistant to fluconazole and itraconazole, were susceptible to low doses of red propolis extracts. These same three specimens had their biofilm formation inhibted by red propolis ethanolic extract.

**Conclusions:**

Thus, red propolis can be faced as a promising natural product to be used in the auxiliary antifungal therapy of denture stomatitis.

**Supplementary Information:**

The online version contains supplementary material available at 10.1186/s12906-021-03445-5.

## Background

An estimated 8.7 million eukaryotic species live on Earth, of which fungi are approximately 7% [1], and about 600 fungal species are human pathogens [2]. Fungi can cause oral infections [3, 4], with *Candida spp.* being the most important etiologic agent of oral diseases [4]. Oral candidiasis is an opportunistic infection that develops in the presence of several predisposing conditions, such as immunodeficiencies, endocrine disorders and poor oral hygiene [5]. *C. albicans* is the most frequent agent found in oral candidiasis, but other *Candida* species, such as *C. parapsilosis*, *C. tropicalis*, *C. glabrata*, *C. krusei*, *C. pseudotropicalis* and *C. guilliermondii*, known as non-*Candida albicans* species (NCA), have been isolated from several clinical cases of the disease [4].

Oral candidiasis associated with denture stomatitis are difficult to treat, and high rates of recurrence are reported [6, 7]. The most common treatment for denture stomatitis includes denture hygiene associated with the use of synthetic antifungal drugs [8]. However, the currently available drugs are not fully effective [9], as shown by the occurrence of resistant strains, infection relapse due to the unappropriated use of drugs and the persistence of the fungal infection even after treatment [10]. Furthermore, *Candida* spp. is known to be a competent biofilm-forming microorganism, and this situation is correlated with an enhanced resistance to antifungals [11–13]. The formation of biofilm by *Candida* spp. is considered a factor that contributes to the recurrence of oral candidiasis and development of chronic infections [14]. In this context, the antimicrobial activity of natural derivatives, such as propolis, has been seen as a promising new therapeutic strategy [7].

Propolis has antibacterial [15], anti-caries [16], anti-inflammatory [17], antioxidant [4], antifungal [18], immunomodulatory [19], anticancer and antiproliferative properties [20]. More than 300 different components were identified in samples of propolis from different origins [21]. In Brazil, 14 different types of propolis were classified based on their geographic origin, color, and physicochemical properties [22–24]. The traditional methods for obtaining or fractioning propolis extracts are distillation (with or without vacuum), extraction by liquid solvents, chromatography, adsorption, and membrane-selective processes [25]. Among these, ethanol is commonly used as a consequence of its chemical affinity to several propolis compounds with important biological activities [26]. With the objective to improve the concentration of bioactive compounds in propolis extracts and to enhance the reproducibility of the extraction method, the use of ultrasound as a pretreatment of the extraction process has been proposed [27]. Both et al. [28] found that the use of an ultrasound-assisted technology for the extraction of polyphenols from black tea enhanced the yield of this compound by 15%.

Considering the need for more accurate treatments for oral candidiasis, the increasing resistance profile of *Candida* spp. strains and the promising use of ultrasound-assisted extraction technologies, the present study aimed to compare the antifungal activity of green and red propolis extracts obtained with and without ultrasound as an extraction pretreatment against oral *Candida* isolates.

## Methods

### Fungal samples and ethical aspects

The clinical isolates used in this study were obtained from patients screened by dentistry professionals at the Dentistry Ambulatory of the UNIME University, Salvador, Brazil. The samples were collected from palate denture stomatitis lesions suggestive of oral candidiasis using sterile swabs, inoculated on Sabouraud dextrose agar (SDA) (HIMEDIA, Mumbai, India) supplemented with 0.2% chloramphenicol, and incubated at 37 °C for 24–48 h [29]. The colonies were then isolated and maintained by weekly reinoculations in the same media. As references, we used four *C. albicans* strains kindly supplied by the Fundação Oswaldo Cruz (FIOCRUZ - IOC 2508, IOC 2517, IOC 3703 and IOC 3704).

The Ethics Committee of the Institute of Health Sciences, Federal University of Bahia, Salvador, Brazil (protocol number 2.118.563) approved this research.

### Identification of the fungal isolates

All clinical isolates were inoculated in CHROMagar™ Candida® for presumptive differentiation of the *Candida* species as described by Madhavan et al. [30]. To confirm the identifications, DNA sequencing of ITS and nuclear large subunit rDNA (LSU) regions was carried out. Briefly, the extraction of the genomic DNAs of the *Candida* spp. clinical isolates was performed using the FastDNA Spin Kit (Mp Biomedicals, Solon, OH, USA). Polymerase chain reactions (PCR) were performed using the primers ITS4 and ITS5 for the amplification of the complete internal transcribed region 26, and LROR and LR7 for the amplification of the LSU region (Mycology Lab – Duke University | Duke Mycology, 2019) [31]. All PCR reactions were performed using Quatro G Taq DNA polymerase (Porto Alegre, RS, Brazil) in a final volume of 50 μL, containing 10 μl of Quatro G buffer, 3.0 μl MgCl 2 (50 mM), 1 μl DNTP (10 mM), 1 μl forward primer (10 pmol), 1 μl reverse primer (10 pmol), 1 μl DMSO, 1.5 μl BSA (1 μg/μL), 5 μL betaine (5 M), 0.2 μL Taq 5 U/μL), 24.8 μL sterile water and 1 μL DNA template. The reactions were carried out in thermocycler as following: 2 min at 94 °C, 35 cycles of 1 min at 94 °C, 1 min at 55 °C, 1 min at 72 °C, and a final extension of 5 min at 72 °C.

Subsequently, the ethanol/EDTA 125 mM precipitation protocol was used to obtain the purified PCR products. The DNA sequencing was executed on 3130xl automated sequencer (Applied Biosystems, Life Technologies, Carlsbad, CA, USA). Consensus sequences were submitted to the Basic Local Alignment Search Tool (BLAST) for the species identification, based on the similarity analysis with nucleotide sequences from the National Center for Biotechnology Information (NCBI) GenBank database.

### Propolis samples

The red propolis was collected in the state of Bahia, Brazil, and the green propolis was collected in the state of Minas Gerais, Brazil. Four different propolis extracts were used in this experiment. The ethanolic green and red propolis extracts were obtained using conventional methods [20, 22]; briefly, frozen red and green propolis were crushed and sieved (60 mesh), with a final particle size of approximately 0.250 nm, and homogenized 2 g samples of each propolis were extracted with ethanol (15 mL, 80%) by mixing the samples for 30 min under constant agitation in an incubation shaker (MA 420/MARCONI—Brazil) at 70 °C and 710 rpm. The extract was recovered by centrifugation for 11 min at 8800 rpm and 5 °C. Then, an additional centrifugation step was performed with 10 mL of ethanol (80%). The supernatant was collected, homogenized, and kept at 50 °C until completely dry. Afterwards, the extracts were stored in tubes, wrapped in aluminum foil at inert atmospheric conditions (N_2_) to avoid degradation. All extracts were kept at 5 °C until use. The other two extracts were obtained using the same method, but with the inclusion of a pretreatment with ultrasound at 50 °C for 20 min, according to Reis and collaborators [32]. The quantification of p-coumaric acid, artepilin C, formononetin and kaempferol in the extracts was performed using a high-performance liquid chromatography (HPLC) system equipped with an automatic injector and diode array detector (DAD). The content of total phenolic compounds was achieved based on the reaction with the Folin-Ciocalteau reagent, followed by spectrophotometry analysis at 765 nm. The content of total flavonoid compounds was determined using a method based on the reaction with a 2% methanol solution of aluminum chloride, along with a quercetin standard curve (5 to 105 μg/mL), followed by spectrophotometry analysis at 415 nm. These results, as defined by previous studies [20, 32], are shown at the Supplementary Table 1. All the propolis samples were obtained from the *Apis mellifera* bee species.

### Broth microdilution assay

The fungicidal activity of commercial antifungal drugs and Brazilian green and red propolis extracts were evaluated using the broth microdilution assay, as described by the M27-A3 protocol from the Clinical Laboratory Standards Institute [33]. Briefly, the yeasts were resuspended in sterile 0.9% saline solution and adjusted by spectrophotometry to a 0.8–1.0 optical density (530 nm), which corresponds to 0.5 on the McFarland scale. Subsequently, the yeasts were diluted 1:50 in sterile saline solution and then diluted 1:20 in RPMI 1640 culture medium (Sigma Aldrich, St Louis, MO, USA) supplemented with 2% glucose [34] for the inoculum obtaining [33].

The four different propolis extracts used in this study, green propolis extract without ultrasound pretreatment (GP_EtOH), green propolis extract with ultrasound pretreatment (GP_US), red propolis extract without ultrasound pretreatment (RP_EtOH) and red propolis extract with ultrasound pretreatment (RP_US), were dissolved in dimethyl sulfoxide (DMSO) and then serially diluted in 1% DMSO (concentration ranging from 0.015625 to 8 mg/mL). Commercial antifungal agents were used as reference drugs, as follows: fluconazole, with concentrations ranging from 0.125 to 64 μg/mL; itraconazole, ketoconazole, nystatin and amphotericin B, with concentrations ranging from 0.0313 to 16 μg/mL.

The inoculum was added to 96-well sterile culture plates at 100 μL per well, followed by the addition of the commercial fungicides and propolis extracts in different concentrations. As a negative control, it was used pure RPMI 1640 media with propolis extracts and antifungals at different concentrations, but without the inoculum. As a positive control, culture media with the fungal inoculum and without any treatment was used. The plates were then incubated for 48 h at 37 °C. Then, the *Candida* spp. growth was assessed using a spectrophotometer (Thermo Scientific, USA) at 625 nm. Each combination of inoculum and propolis or fungicide treatment was performed in triplicate, and the entire procedure was repeated twice.

The minimum inhibitory concentration (MIC) value, which represented the lowest concentration that inhibited 100% of the fungal growth, was determined by the minimum concentration of propolis or antifungal that resulted in no optic densitometry (OD) reading above the negative control OD value. For the determination of the minimum fungicide concentration (MFC - minimal drug concentration able to kill 100% of the yeasts), aliquots from each well of the broth microdilution assay were plated in SDA and then incubated at 37 °C for additional 48 h. Thus, the lowest concentration that revealed no visible fungal growth was determined as the MFC. According to the M27-S4 document [35], the breakpoint for fluconazole was considered for the classification of all isolates according to the corresponding MIC (at μg/mL) as follows: resistant (R) ≥ 8; dose-dependent susceptible (SDD) = 4; susceptible (S) ≤ 2. For itraconazole, it was considered the M27-A3 document classification [33].

### Biofilm interference assay

To evaluate the propolis activity against biofilm formation, the ethanolic red propolis extract (RP_EtOH) was tested against three clinical isolates: *C. albicans* PAC 8, *C. dubliniensis* PAC 1, and *C. tropicalis* PAC 15. These species were chosen because of its different susceptibility to RP_EtOH, as determined by the microdilution test. The ability of RP_EtOH to inhibit biofilm formation was evaluated as previously described [36]. Briefly, the strains were incubated in Sabouraud Dextrose Broth at 37 °C in a shaker at 250 rpm for 15 h. The cultures had the cell density adjusted to an OD_600_ of 0.38–0.5 with RPMI media and then added to a 96 well plate. The plate was incubated in a shaker at 250 rpm at 37 °C for 90 min. After that time, the media was aspirated, the wells were washed with PBS and RP_EtOH diluted in RPMI (concentrations ranging from 1 to 16 mg/mL) was added. 24 h later, the optical density was read in a spectrophotometer at 570 nm. The interference rates in % were obtained using the following formula [37]:

$$[(\mathrm{OD}_{570}\;\mathrm{of}\; {Candida}\; \mathrm{spp.} \;\mathrm{Treated}\; \mathrm{with} \;\mathrm{RP}\_\mathrm{EtOH} * \mathrm{100}) / \mathrm{OD}_{570} \;\mathrm{of}\;\mathrm{non-treated} \;{Candida} \;\mathrm{spp.}] - 100$$

## Results

### Identification of the *Candida* species isolated from denture stomatitis cases

The twelve oral cavity *Candida* clinical isolates were phenotypically identified using CHROMagar™ *Candida* (data not shown) and Sanger sequencing (Tables 1 and 2). Our results showed the presence of three *Candida* species with different frequencies. *C. albicans* was the most frequent isolated species (58.33% - 7/12), followed by *C. tropicalis* (33.33% - 4/12) and *C. dubliniensis* being the less abundant of the isolated species (8.33% - 1/12).

**Table 1:** MIC and MFC values obtained for *Candida* spp. reference strains and clinical isolates incubated with commercial fungicides (fluconazole, ketoconazole, itraconazole, nystatin and amphotericin B). *Candida* spp. strains and isolates were incubated with different concentrations of the fungicides, as suggested by the M27-A3 protocol from the CLSI (2008). MIC - Minimal Inhibitory Concentration; MFC - Minimal Fungicidal Concentration; (S) - Susceptible; (S-DD) - Dose-dependent Susceptibility; (R) - Resistant

Isolate	Species	Fluconazole		Ketoconazole		Itraconazole		Nystatin		Amphotericin B	
		(μg/mL)		(μg/mL)		(μg/mL)		(μg/mL)		(μg/mL)	
		MIC	MFC	MIC	MFC	MIC	MFC	MIC	MFC	MIC	MFC
2508	*C. albicans*	0.125 (S)	0.125	0.03125	0.03125	0.03125 (S)	0.03125	4	4	1	1
2517	*C. albicans*	0.25 (S)	0.25	0.03125	0.03125	0.125 (S)	0.0125	4	8	1	1
3703	*C. albicans*	0.125 (S)	0.125	0.03125	0.03125	0.03125 (S)	0.03125	4	4	0.5	0.5
3704	*C. albicans*	0.5 (S)	0.5	0.03125	0.0625	0.03125 (S)	0.03125	2	4	0.5	0.5
PAC 01	*C. dubliniensis*	32 (S-DD)	> 64	0.03125	0.125	8 (R)	> 16	2	4	2	2
PAC 02	*C. tropicalis*	2 (S)	16	0.03125	0.125	0.25 (S-DD)	0.5	2	4	2	2
PAC 04	*C. tropicalis*	0.125 (S)	0.5	0.03125	0.03125	0.03125 (S)	0.03125	0.5	2	0.25	0.5
PAC 05	*C. tropicalis*	8 (R)	> 64	0.25	> 16	1 (R)	> 16	2	4	2	4
PAC 06	*C. albicans*	0.25 (S)	2	0.0625	0.125	0.0625 (S)	0.5	4	4	2	2
PAC 08	*C. albicans*	1 (S)	8	0.03125	0.125	0.25 (S-DD)	2	2	4	2	2
PAC 13	*C. albicans*	0.5 (S)	16	0.03125	> 16	0.0625 (S)	0.25	1	4	0.5	2
PAC 15	*C. tropicalis*	2 (S)	> 64	0.03125	> 16	2 (R)	8	1	4	2	2
PAC 17	*C. albicans*	16 (R)	> 64	0.03125	> 16	2 (R)	> 16	16	> 16	4	4
PAC 18	*C. albicans*	1 (S)	> 64	0.03125	> 16	0.25 (S-DD)	> 16	16	16	1	2
PAC 19	*C. albicans*	4 (S-DD)	16	0.125	1	1 (R)	8	8	8	0.5	0.5
PAC 20	*C. albicans*	4 (S-DD)	> 64	0.0625	> 16	1 (R)	> 16	4	16	0.25	0.5

**Table 2:** MIC and MFC values obtained for Candida spp. reference strains and clinical isolates incubated with different concentration of four propolis extracts. *Candida* spp. strains and clinical isolates were incubated for 48 h with different concentrations of the four propolis extracts, and the growth inhibition was then calculated. MIC: Minimal Inhibitory Concentration; MFC: Minimal Fungicidal Concentration; GP_EtOH: green propolis ethanolic extract; GP_US: green propolis ethanolic extract pre-treated with ultrasound; RP_EtOH: red propolis ethanolic extract; RP_US: red propolis ethanolic extract pre-treated with ultrasound

Isolate	Species	GP_EtOH (mg/mL)		GP_US (mg/mL)		RP_EtOH (mg/mL)		RP_US (mg/mL)	
		MIC	MFC	MIC	MFC	MIC	MFC	MIC	MFC
2508	*C. albicans*	2	4	4	> 8	1	2	2	2
2517	*C. albicans*	4	4	2	2	0.5	1	1	2
3703	*C. albicans*	4	8	4	> 8	0.5	4	2	4
3704	*C. albicans*	4	8	2	4	0.5	2	0.25	2
PAC 01	*C. dubliniensis*	2	4	2	> 8	1	2	2	2
PAC 02	*C. tropicalis*	4	> 8	2	2	1	4	0.015	2
PAC 04	*C. tropicalis*	1	4	1	2	0.125	1	1	2
PAC 05	*C. tropicalis*	4	> 8	8	> 8	1	2	2	2
PAC 06	*C. albicans*	> 8	> 8	> 8	> 8	4	> 8	2	> 8
PAC 08	*C. albicans*	> 8	> 8	> 8	> 8	> 8	> 8	2	> 8
PAC 13	*C. albicans*	> 8	> 8	4	> 8	4	4	1	> 8
PAC 15	*C. tropicalis*	> 8	> 8	> 8	> 8	4	> 8	2	2
PAC 17	*C. albicans*	> 8	> 8	> 8	> 8	2	> 8	1	2
PAC 18	*C. albicans*	> 8	> 8	> 8	> 8	2	> 8	2	2
PAC 19	*C. albicans*	> 8	> 8	> 8	> 8	1	1	0.015	0.125
PAC 20	*C. albicans*	> 8	> 8	4	> 8	1	2	1	2

### Susceptibility to the commercial antifungal drugs

The results showed that the reference strains and the clinical isolates present distinct resistance patterns for the three azoles antifungal drugs used in this study (fluconazole, ketoconazole and itraconazole). The four *C. albicans* reference strains were all susceptible to fluconazole, with MIC ranging from 0.125 to 0.5 μg/mL. The results of *C. albicans* susceptibility to fluconazole showed that four (4/7) isolates were susceptible (MIC ≤2 μg/mL), two (2/7) were dose-dependently susceptible (S-DD) (MIC = 4 μg/mL) and one (1/7) was resistant (MIC = 16 μg/mL) (Table 1). Thus, 14.28% of the *C. albicans* clinical isolates presented resistance to fluconazole.

The evaluation of all *Candida* spp. oral isolates and reference strains used in this study showed that *C. albicans* had a great variability for fluconazole susceptibility, with MIC values ranging from 0.25 to 16 μg/mL. Eight *C. albicans* and three *C. tropicalis* were susceptible to fluconazole (MIC ≤2 μg/mL); one *C. albicans* and one *C. tropicalis* were resistant (MIC ≥8 μg/mL); and two *C. albicans* (MIC = 4 μg/mL) and one *C. dubliniensis* (MIC = 32 μg/mL) were fluconazole S-DD. The fluconazole MFCs for *C. albicans* were markedly variable, ranging from 0.125 to undetermined values (> 64 μg/mL). Eight of all *C. albicans* (8/11) showed MFC ranging from 0.125 to 16 μg/mL, and three (3/11) could not be determined (> 64 μg/mL). Considering the NCA species, the MFC for the *C. dubliniensis* isolate could not be determined (MFC > 64 μg/mL). Finally, *C. tropicalis* fluconazole MFC was determined for two isolates (MFC = 0.5 and 16 μg/mL) and undetermined for the other two isolates (> 64 μg/mL) (Table 1).

The fungistatic effect of ketoconazole was observed at low concentrations for all *C. albicans* strains (MIC = 0.03125 to 0.125 μg/mL). However, the MFC was undetermined for four (4/11) of the *C. albicans* specimens (MFC > 64 μg/mL), being all of them clinical isolates. Regarding the other seven (7/11) *C. albicans,* four reference strains and three clinical isolates presented MFC ranging from 0.03125 to 1 μg/mL. *C. dubliniensis* presented ketoconazole MIC of 0.03125 μg/mL and MFC of 0.125 μg/mL. Regarding the *C. tropicalis* isolates*,* the MIC were 0.03125 and 0.25 μg/mL, and MFC were found for two isolates, with values of 0.03125 and 0.125 μg/mL, but it could not be determined (MFC > 16 μg/mL) for the other two isolates (Table 1). Since the M27-S4 document [35] do not provide a ketoconazole susceptibility classification, this specific classification could not be performed to this drug in this study.

Of the three azoles drugs tested in this study, itraconazole was the one that it could be observed a higher number of resistant isolates. Of the 16 *C. albicans* specimens, four reference strains and 12 clinical isolates, only seven (7/16) were susceptible (MIC ≤0.125 μg/mL), and one of the four *C. tropicalis* was susceptible to this drug (MIC = 0.03125 μg/mL). Of the remaining *Candida* specimens tested, six isolates were resistant (MIC ≥1 μg/mL): three *C. albicans,* two *C. tropicalis* and the *C. dubliniensis* isolate; two (2/16) *C. albicans* (MIC = 0.25 μg/mL) and one *C. tropicalis* (MIC = 0.25 μg/mL) were S-DD to itraconazole (Table 1). The fungicidal concentration (MFC) of itraconazole could be determinate for eight (8/11) of the *C. albicans* tested, ranging from 0.03125 to 8 μg/mL. For the other three *C. albicans* (3/11) isolates, the MFC could not be determined (MFC > 16 μg/mL), and these three clinical isolates were resistant to itraconazole. For three (3/4) *C. tropicalis* isolates, the fungicidal concentration for itraconazole was determined (ranging from 0.03125 and 8 μg/mL). For one *C. tropicalis* and the *C. dubliniensis* isolate, the MFC could not be determined (MFC > 16 μg/mL).

Nystatin and amphotericin B (AmB) presented the lowest variabilities in the MIC and MFC values for all *Candida* spp. tested in this study. As for itraconazole, nystatin and AmB do not have a susceptibility classification in the M27-S4 document. All *C. albicans* presented MIC between 1 and 16 μg/mL and MFC ranging from 4 to 16 μg/mL for nystatin, except for PAC17 that presented a not determined MFC (> 16 μg/mL). *C. glabrata* had a nystatin MIC of 2 μg/mL and MFC of 4 μg/mL. Regarding the response of *C. tropicalis* to nystatin, the MIC ranged from 0.5 to 2 μg/mL, and the MFC ranged from 2 to 4 μg/mL. Interesting, AmB had equal MIC and MFC values for almost all strains (Table 1). The AmB MIC for *C. albicans* ranged from 0.25 to 4 μg/mL and the MFC ranged from 0.5 to 4 μg/mL. For *C. dubliniensis,* the MIC and the MFC were the same (2 μg/mL). Finally, for *C. tropicalis* isolates, the MFC ranged from 0.25 to 2 μg/mL, and the MFC ranged 0.5 and 4 μg/mL.

When analyzing each isolate alone, it can be seen that the clinical isolate *C. albicans* PAC 17 presented the highest MIC and MFC concentrations for all commercial drugs tested. Thus, this isolate can be considered a potential multidrug resistant organism (Table 1).

### Susceptibility to Brazilian green and red Propolis

**Fig. 1:**
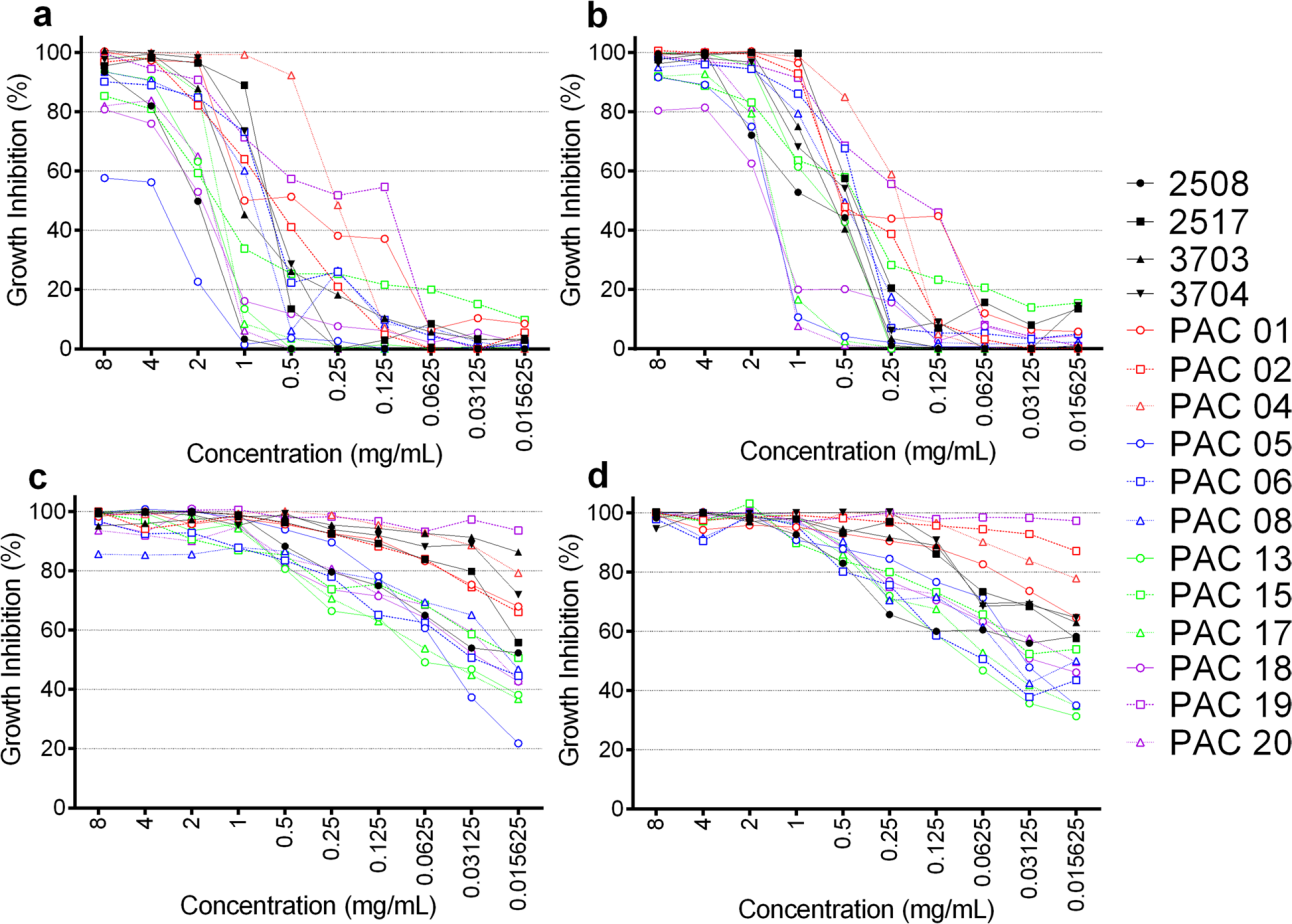
Growth inhibition curves of *Candida albicans* reference strains (2508, 2517, 3703 and 3704) and clinical isolates treated with **(a)** green propolis ethanolic extract, **(b)** green propolis ethanolic extract pretreated with ultrasound, **(c)** red propolis ethanolic extract and **(d)** red propolis ethanolic extract pretreated with ultrasound. Microdilution assays were performed in duplicate. *Candida* spp. isolates were incubated with propolis extracts (concentrations ranging from 0.015 to mg/mL) for 48 h. Then, the relative growth was obtained using a spectrophotometer (625 nm) and the growth inhibition rates were calculated. The results represent the mean of two independent experiments

**Fig. 2:**
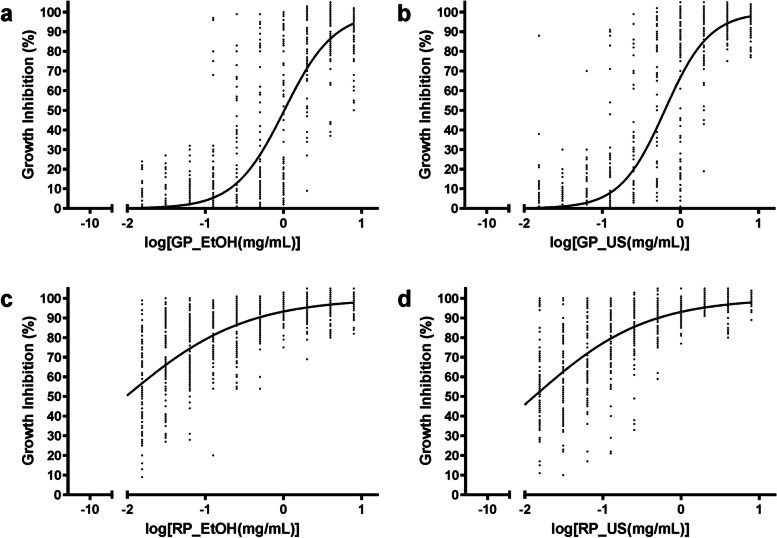
Dose-response curve of *Candida* spp. treated with (**a**) green propolis ethanolic extract, (**b**) green propolis ethanolic extract pretreated with ultrasound, (**c**) red propolis ethanolic extract and (**d**) red propolis ethanolic extract pretreated with ultrasound. Microdilution assays were performed in duplicate. *Candida* spp. were incubated with a range of propolis concentrations (8 to 0.015 mg/mL) for 48 h. Then, the relative growth was obtained using a spectrophotometer (625 nm), and the growth inhibition rates were calculated. Graphical representations and dose-response stimulation statistics were obtained using the software Graph Pad Prism 6

All green and red propolis extracts evaluated herein induced growth inhibition in all *Candida* spp. specimens tested in this study (Fig. 1). However, both red propolis extracts were more effective in lower concentrations when compared to the green propolis extracts (Figs. 1 and 2). The dose-response curves showed that all *Candida* spp. had a higher tolerance to both green propolis extracts in lower concentrations; however, above 0.125 mg/mL, the growth inhibition was ≥50%, and it was above 95% at 8 mg/mL (Fig. 2 - a and b). In contrast, both red propolis extracts, with and without ultrasound pretreatment, presented a higher activity even in the lowest concentration tested (0.015 mg/mL) (Fig. 2 - c and d).

The green propolis extracted with different pretreatments were tested against the reference strains 2508, 2517, 3703 and 3704, and the MIC values of GP_EtOH and GP_US ranged from 2 to 4 mg/mL (Table 2). However, when we evaluated the MFC for GP_EtOH (4 to 8 mg/mL), it could be determined for all reference strains, while for GP_US the MFC (2 to > 8 mg/mL) could not be determined for two (2508 and 3703) of the four reference strains (Table 2). Concerning the clinical isolates, the MIC and MFC were undetermined for most of the *Candida* spp. treated with both green propolis extracts. The percentage of the clinical isolates with undetermined MIC and MFC values were 66.7% (8/12) and 83.3% (10/12) for GP_EtOH and 50% (6/12) and 83.3% (10/12) for GP_US. Only the *C. dubliniensis* and three *C. tropicalis* (3/4) isolates had a defined MIC for GP_EtOH, with values ranging from 1 to 4 mg/mL. Regarding MFC values, only the *C. dubliniensis* (MFC = 4 mg/mL) and one of the *C. tropicalis* clinical isolates (MFC = 4 mg/mL) presented a defined MIC for GP_EtOH. The response to GP_US by the clinical isolates showed two *C. albicans* (2/7), the *C. dubliniensis* isolate and the three *C. tropicalis* (3/4) with defined MICs, with values ranging from 1 to 8 mg/mL. When considering these specific isolates, only two *C. tropicalis* isolates presented determined MFC values (2 mg/mL) (Table 2).

For the red propolis extracts (Table 2), the MIC values were 0.5 and 1 mg/mL for RP_EtOH and 0.25 to 2 mg/mL for RP_US, when considering the reference strains. For these same strains, the MFC ranged from 1 to 4 mg/mL for RP_EtOH, and 2 to 4 mg/mL for RP_US. The *Candida* spp. clinical isolates presented a large variability in the MIC and MFC values for both red propolis extracts evaluated. Only 8.3% (1/12) of clinical isolates tested with RP_EtOH presented an undetermined MIC (> 8 mg/mL). This percentage corresponded to juts one *C. albicans* isolate (PAC 08). The other *C. albicans* clinical isolates (6/7) presented MIC values between 1 and 4 mg/mL for RP_EtOH. Of these *C. albicans* isolates, only three (3/7) had MFC defined values ranging from 1 to 4 mg/mL for RP_EtOH. The *C. dubliniensis* isolate had a MIC of 1 mg/mL and MFC of 2 for RP_EtOH. *C. tropicalis* isolates presented a MIC ranging from 0.125 to 4 mg/mL, and MFC from 1 to 4 mg/mL for three of them, and undefined for one isolate (MFC > 8 mg/mL) **(**Table 2**)**. For RP_US, all MIC values could be determined below 8 mg/mL, ranging between 0.015 to 2 mg/mL for the clinical isolates. Interesting, the *C. albicans* PAC 19 presented the lowest values for MIC and MFC for RP_US. A MFC > 8 was observed in 41.7% (5/12) and 25% (3/12) of the clinical isolates for RP_EtOH and RP_US, respectively.

An interesting finding of this study was the susceptibility to the red propolis extracts of the clinical isolate PAC 05 (*C. tropicalis*), PAC 17 and PAC 19 (*C. albicans*) isolates*,* since they presented resistance to fluconazole and itraconazole (Tables 1 and 2). Specifically, the clinical isolate *C. albicans* PAC 17, besides presenting resistance to the azoles herein included, also presented high MIC and MFC values for the other commercial drugs tested herein (Table 1).

### Biofilm formation interference by red Propolis

**Fig. 3:**
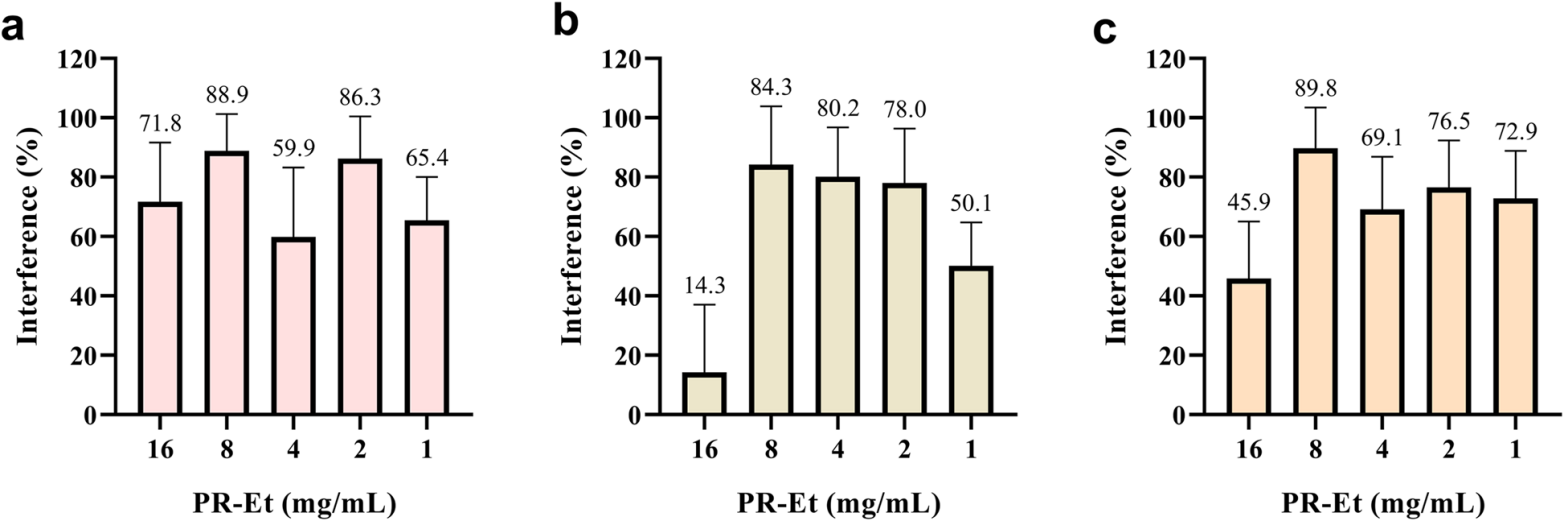
Interference (%) of red propolis ethanolic extract in the biofilm formation by (**a**) *C. albicans* (PAC 08), (**b**) *C. dubliniensis* (PAC 01) and (**c**) *C. tropicalis* (PAC 15). The experiment was performed in quadruplicate. The graphical representation was obtained using the software GraphPad Prism 6

The red propolis ethanolic extract was able to interfere in the biofilm formation by all the three strains tested in this specific assay (Fig. 3a, b and c). In all cases, the concentration of 8 mg/mL induced the highest interference values, reaching the maximum of 89.8%. The concentration of 16 mg/mL caused interferences in the readings by the spectrophotometer since it condensed in the bottom of the wells. *C. dubliniensis* (PAC 1) (Fig. 3 – b) was the least susceptible isolate when compared to the other ones tested in the assay, but even so, its biofilm formation was reduced by 78% at the concentration of 2 mg/mL. Regarding the other two strains – *C. albicans* (Fig. 3a) and *C. tropicalis* (Fig. 3c), the treatment with the red propolis ethanolic extract at the lowest concentration tested (4 mg/mL) presented interference results of 65.4 and 72.9%, respectively.

## Discussion

Denture stomatitis is an infection of the oral cavity characterized by inflammation and erythema, being the fungi from the *Candida* genus important etiologic agents of the disease [38]. The presence of *Candida albicans* and *Candida*-non *albicans* in these infections have been already described [39, 40]. The identification of these species is usually based on the use of a chromogenic medium which has been described as a good accurate method [30, 41]. However, the molecular approach represents a more accurate method for species identification [42]. Our results showed the presence of three *Candida* species isolated from denture stomatitis lesions, being *C. albicans* the most frequent species and, in a lower abundance, *C. tropicalis* and *C. dubliniensis*. *C. albicans* and *C. tropicalis* species were found with a similar frequency causing chronic periodontitis at Alagoas [3], a Brazilian state close to Bahia state, where this study was conducted. In Pará state, Brazil, *C. albicans* was also the most frequent species associated with this oral disease, with a 78% frequency; additionally, *C. tropicalis*, *C. famata* and *C. parapsilosis* were also found, but in association with *C. albicans* at the infection site [39]. In Spain, *C. albicans* corresponded to 70% of *Candida* specimens isolated from oral candidiasis cases, followed by *C. glabrata* (8.6%), *C. parapsilosis* (7.4%) and *C. tropicalis* (3.3%) [41].

The main drugs used to treat denture stomatitis are miconazole, fluconazole, itraconazole, nystatin, amphotericin B, ketoconazole, clotrimazole and chlorhexidine [43]. In this way, this study used five important commercial drugs that are routinely used for oral candidiasis treatment. Our results showed a great variability in *C. albicans* susceptibility to fluconazole. Besides this, most of the isolates were susceptible to this drug. Siqueira and collaborators [3] showed that some clinical oral isolates of *C. albicans* presented a great variability in fluconazole susceptibility, and the occurrence of resistance in 36.8% of the *Candida* spp. isolates tested. In yeasts isolated from bloodstream infections, fluconazole susceptibility tests have demonstrated that 19% of the isolates were resistant to fluconazole [44]. In addition, the susceptibility test to antifungal agents against *C. albicans* and NCA isolates, when using the criteria of the CLSI document M27-S4, has shown an 2.4-fold increase in the number of species not susceptible to these drugs, when compared to the criteria of document M27-A3 [45].

Regarding the NCA species studied herein and its susceptibility profiles to fluconazole and itraconazole, different patterns were observed. Fluconazole resistance is more common in NCA species than in *C. albicans* [44, 46]. Despite this, no correlation between the phylogenetic distribution of *Candida* isolates and the susceptibility profile fluconazole has been found [47]. Omran et al. [40] observed that *C. albicans*, *C. glabrata* and *C. tropicalis* isolated from Iranian patients with denture stomatitis presented 15.5, 17.4 and 12.5% of in vitro resistance to fluconazole, respectively. As other NCA species, *C. dubliniensis* was associate to infections in immunocompromised patients [48, 49]. *C. dubliniensis* azoles resistance cases were already demonstrated in few studies [50, 51]; an in vitro induction of fluconazole-resistance showed that *C. dubliniensis* can easily develop resistance to this drug [50]. Nevertheless, the increase in fluconazole-less susceptible or resistant C*andida* strains indicates that the use of alternative drugs in the treatment of oral candidiasis is urgently needed [9, 10].

The results obtained in ketoconazole susceptibility assays showed lower MIC values. However, the MFC evaluation showed that this drug failed to present a fungicidal effect against the *Candida* spp. clinical isolates. Ernst and collaborators [52] showed that fluconazole was fungistatic against *C. albicans* and *Candida neoformans,* but without measurable fungicidal activity. For *Trichosporon asahii*, azoles showed a fungistatic effect, but without fungicidal activity [53]. The absence of a fungicidal effect can lead to the selection of resistant lines and consequent treatment failure, increasing the number of relapse cases [54].

Our data showed a great variability in the nystatin MIC and MFC values. However, different to what were observed for the azoles, all MIC values for nystatin and Amphotericin B (AmB) could be determined and, except for one *C. albicans* isolate for nystatin, all isolates had their MFC values determined. Nystatin and AmB are antifungals from the polyene class and its activity is associated to the membrane ergosterol content [54]. Miranda-Cadena et al. [41] showed that nystatin presented an excellent antifungal activity against all tested isolates, in opposition to fluconazole and itraconazole. Regarding AmB, our results still showed similar MIC and MFC values in almost all *Candida* isolates. In a previous study, *C. albicans* presented 71% agreement between MIC and MFC values after exposure to AmB [55]. This situation suggests that the same concentration of the drug is able to inhibit the growth and to kill the fungi, as well as facilitate the treatment of the disease, since the drug concentration can be more effective in the control of the fungal spread.

The evaluation of the Brazilian green and red propolis extracts dose-response curves indicated that both red propolis extracts are potent agents against *Candida* spp. when compared to the green propolis extracts. The antimicrobial activity of the propolis extracts is attributed to the phenolic and flavonoid content [17, 20]. Several researchers have reported that different propolis extracts present antifungal [18], antimicrobial [18, 56], antitumoral [57], antioxidant [58], anti-inflammatory and immunomodulatory properties [17, 59]. Green propolis extract activities have been associated in part to artepillin C, a cinnamic acid derivative present in high amounts in Brazilian green propolis samples [20, 58]. Artepillin C is considered one of the main active components of the green propolis extracts and exhibits antitumor [60], anti-inflammatory [61] and antimicrobial activities [62].

The chemical composition of the green and red propolis ethanolic extracts [20, 32] and of the red and green propolis ultrasound-assisted extracts used in this study [32], focused in compounds that have already been cited as having antimicrobial activities, are shown at the Supplementary Table 1. The isoflavonoid formononetin was found in the in Brazilian green propolis ethanolic extract at the concentration of 4 mg/g [20], while red propolis ethanolic extracts presented 8.68 mg/g of this compound [32]. For propolis extracts submitted to ultrasound pre-treatment, formononetin was present at concentrations of 7.77 mg/g in green propolis and 8.40 mg/g in red propolis [32]. Red propolis ethanolic extracts showed almost two times more formononetin when compared to green propolis ethanolic extracts, while the green and red propolis ultrasound-treated extracts present almost the same formononetin concentration. This compound is considered one of the major active components of the red propolis extract due to its diverse biological activities [18, 20, 63–65]. Thus, the high formononetin content of the red propolis can be associated to the best *Candida* growth inhibition by red propolis when compared to green propolis, and this situation can be supported by the results presented by the treatment with the ultrasound-treated extracts.

Regarding total phenolic compounds and flavonoids, both green and red propolis ethanolic extracts demonstrated to have high contents of these compounds. Green propolis ethanolic extracts showed a concentration of 181.71 mg EAG/g for total phenolic compounds [20] and 46.80 mg EQ/g of flavonoids [20], while ultrasound treated green propolis extracts had 342.09 mg EAG/g and 22.68 mg EQ/g of total phenolic compounds and flavonoids, respectively. Red propolis ethanolic extracts presented concentration of 308.49 mg GAE/g of total phenolic compounds and 82.87 mg EQ/g of flavonoids, while ultrasound-treated red propolis extracts presented 314.75 mg GAE/g of total phenolic compounds and 90.38 mg EQ/g of flavonoids [32]. The green propolis extract obtained using ultrasound-assisted extraction has more total phenolic compounds than the ethanolic extract. Red propolis do not showed any differences in total phenolic compound concentration in the extracts obtained by both extraction methodologies. However, when the flavonoids content was compared, the green propolis ethanolic extract has more of this compound than the ultrasound-assisted extract. Red propolis, independently of the extraction method used, had higher contents of flavonoids when compared to green propolis, presenting two and four-fold more flavonoids than green propolis ethanolic and ultrasound extracts, respectively. Propolis antifungal activity have been associated to the high flavonoid content found in different propolis extracts [66, 67]. Considering that the antifungal activity of red propolis extracts were superior to the green propolis extracts in our study, we can associate this better result to the higher contents of flavonoids and formononetin of red propolis.

The comparison between green and red propolis extract activity against *Candida* spp. showed that both red propolis extracts had more fungistatic and fungicidal activity than the green propolis extracts. The scientific literature reports a strong red and green propolis antifungal activity against *Candida* spp. [18]. A variability in antimicrobial and antitumoral activities of colored propolis extracts have already been described, but the green and red propolis showed better results when compared to the yellow or brown propolis extracts [20, 68].

The ultrasound pretreatment in red propolis was able to enhance the final concentration of the isoflavones formononetin (60% increase) and kaempferol (undetectable in red propolis extracts without ultrasound pretreatment), when compared to extracts without this pretreatment [32]. However, even with these higher concentrations of antimicrobial compounds in RP_US, our results showed that just one strain that had MIC > 8 mg / mL when treated with RP_EtOH presented a MIC of 2 mg / mL after exposure to RP_US, and the MFC values of the RP_US presented two fewer undetermined results. This situation can be explained based on the study by Neves et al. [69], where it was found that the acetate fraction of red propolis extracts presented an increased concentration of formononetin, but these fractions did not show a higher fungicidal activity against *Candida* spp. These authors suggested that the antimicrobial activity of red propolis extracts cannot be attributed to just one compound, but is a result of a synergistic effect of them [70].

Our data also showed fungistatic and fungicidal activities of the red propolis extracts against *Candida* spp. that were resistant to fluconazole and itraconazole. In another study, some NCA strains from Rio Grande do Sul, Brazil, selected in a fluconazole resistance step-by-step assay, showed a cross-resistance to itraconazole and an increase in the MIC for ketoconazole, but no change in the susceptibility to red propolis extracts [69]. A clinical trial using a gel and a mouthwash containing green propolis in the treatment of denture stomatitis showed that these treatments had the same effectiveness against *Candida* spp.-associated denture stomatitis as a miconazole gel [8]. Pippi and collaborators [71] demonstrated that the red propolis from Minas Gerais State, Brazil, had a synergic effect with fluconazole, increasing up to 16-fold the fluconazole susceptibility of *C. parapsilosis*, *C. glabrata* and a polymicrobial culture formed by a mix of *C. parapsilosis*, *C. glabrata*, *C. krusei* and *C. tropicalis*; all of them resistant to fluconazole.

The ability of propolis to interfere with the consolidation and adherence of the biofilm of Candida species is poorly investigated. However, Tobaldini-Valerio and collaborators [72] observed that green propolis effectively affected the biofilm formation by *C. albicans, C. parapsilosis* and *C. tropicalis,* but in a species- and strain-dependent manner. In another study, similar results were obtained for green propolis, and the biofilm formation by *C. albicans* was significantly more reduced when compared to *C. parapsilosis* and *C. tropicalis* [73]. In our work, the majority of the RP_EtOH concentrations tested were able to reduce biofilm formation (more than 80%) in the three species tested herein. Most studies had tested the ability of propolis to act on the already formed biofilm [73–75], but as the CDC advises [76], the best way to fight resistant infections is through prevention. Thus, propolis can be used to prevent colonization on abiotic surfaces and in infections by resistant *Candida* spp. Moreover, as previously discussed, considering the increase in resistance against commercial antifungal, all these findings indicate that red propolis extract is a promising candidate for the development of an oral candidiasis auxiliary treatment.

## Conclusions

The results herein presented showed higher antifungal activity by red propolis extracts, when compared to green propolis extracts. Moreover, red propolis had a fungistatic and fungicidal effect on clinical isolates of *C. albicans* and NCA that were resistant to fluconazole and itraconazole, showing its potential use as an auxiliary/adjuvant treatment for oral. Additionally, the ultrasound pretreatment did not improve the antimicrobial activity against *Candida* spp. Regarding biofilms assays, the ethanolic extract of red propolis was able to reduce the formation of biofilm by commercial antifungal resistant *Candida* spp. isolates.

## Supplementary Information

**Additional file 1.**

## Data Availability

All data generated or analyzed during this study are included in this published article and its supplementary information files.

## Abbreviations

AmB: Amphotericin B
BLAST: Basic Local Alignment Search Tool
DMSO: Dimethyl Sulfoxide
SDD: Dose-Dependent Susceptible
FIOCRUZ: Fundação Oswaldo Cruz
GP_US: Green Propolis extract with ultrasound pretreatment
GP_EtOH: Green Propolis extract without ultrasound pretreatment
MFC: Minimal Fungicide Concentrations
MIC: Minimal Inhibitory Concentrations
NCBI: National Center for Biotechnology Information
NCA: Non-*Candida albicans* species
OD: Optic Densitometry
PAC: Pacient
PCR: Polymerase Chain Reactions
RP_US: Red Propolis extract with ultrasound pretreatment
RP_EtOH: Red Propolis extract without ultrasound pretreatment

## References

[1] Mora C, Tittensor DP, Adl S, Simpson AGB, Worm B. How Many Species Are There on Earth and in the Ocean? (2011) PLoS Biol. 9:e1001127. 10.1371/journal.pbio.1001127. 10.1371/journal.pbio.1001127 PMC3160336 21886479

[2] Brown GD, Denning DW, Levitz SM (2012). Tackling human fungal infections. Science.

[3] Siqueira ABS, Rodriguez LRN de A, Santos RKB, Marinho RRB, Abreu S, Peixoto RF, Gurgel BCV. Antifungal activity of propolis against Candida species isolated from cases of chronic periodontitis. Braz Oral Res. 2015;29:1–6. 10.1590/1807-3107BOR-2015.vol29.0083. 10.1590/1807-3107BOR-2015.vol29.0083 26154370

[4] Gleiznys A, Zdanavičienė E, Žilinskas J, Eglė DDS (2015). Juozas Žilinskas DDS (2015) Candida albicans importance to denture wearers. A literature review. Balt Dent Maxillofac J.

[5] Regezi JA (2017). Ciubba, James J. Jordan RCK. Patologia Oral correlações clinicopatológicas. 7 th.

[6] Batista JM, Birman EG, Cury AE (1999). Suscetibilidade a antifúngicos de cepas de Candida albicans isoladas de pacientes com estomatite protética. Rev Odontol da Univ São Paulo.

[7] Leite DP, Piva MR, Martins-Filho PRS (2015). Identificação das espécies de Candida em portadores de estomatite protética e avaliação da susceptibilidade ao miconazol e à terapia fotodinâmica. Rev Odontol da UNESP.

[8] Capistrano HM, de Assis EM, Leal RM, Alvarez-Leite ME, Brener S, Bastos EMAF (2013). Brazilian green Propolis compared to Miconazole gel in the treatment of Candida-associated denture stomatitis. Evidence-Based Complement Altern Med.

[9] Garcia-Cuesta C, Sarrion-Perez M, Bagan J (2014). Current treatment of oral candidiasis: a literature review. J Clin Exp Dent.

[10] Sanguinetti M, Posteraro B, Lass-Flörl C (2015). Antifungal drug resistance among Candida species: mechanisms and clinical impact. Mycoses.

[11] Mukherjee P, Chandra J (2004). Candida biofilm resistance. Drug Resist Updat.

[12] Pereira R, dos Santos Fontenelle RO, de Brito EHS, de Morais SM (2021). Biofilm of Candida albicans: formation, regulation and resistance. J Appl Microbiol.

[13] Holmes AR, Keniya MV, Ivnitski-Steele I, Monk BC, Lamping E, Sklar LA, Cannon RD (2012). The monoamine oxidase a inhibitor Clorgyline is a broad-Spectrum inhibitor of fungal ABC and MFS transporter efflux pump activities which reverses the azole resistance of Candida albicans and Candida glabrata clinical isolates. Antimicrob Agents Chemother.

[14] Douglas LJ (2003). Candida biofilms and their role in infection. Trends Microbiol.

[15] Chen Y-W, Ye S-R, Ting C, Yu Y-H (2018). Antibacterial activity of propolins from Taiwanese green propolis. J Food Drug Anal.

[16] Bueno-Silva B, Koo H, Falsetta ML, Alencar SM, Ikegaki M, Rosalen PL (2013). Effect of neovestitol–vestitol containing Brazilian red propolis on accumulation of biofilm in vitro and development of dental caries in vivo. Biofouling.

[17] Rufatto LC, dos Santos DA, Marinho F, Henriques JAP, Roesch Ely M, Moura S (2017). Red propolis: chemical composition and pharmacological activity. Asian Pac J Trop Biomed.

[18] Freires IA, Queiroz VCPP, Furletti VF, Ikegaki M, de Alencar SM, Duarte MCT, Rosalen PL (2016). Chemical composition and antifungal potential of Brazilian propolis against Candida spp. J Mycol Med.

[19] Gao W, Wu J, Wei J, Pu L, Guo C, Yang J, Yang M, Luo H (2014). Brazilian green propolis improves immune function in aged mice. J Clin Biochem Nutr.

[20] Machado BAS, Silva RPD, Barreto G de A, Costa SS, Silva DF da, Brandão HN, Rocha JLC, DEllagostin OA, Henriques JAP, Umsza-Guez MA, Padilha FF. Chemical composition and biological activity of extracts obtained by supercritical extraction and Ethanolic extraction of Brown, green and red Propolis derived from different geographic regions in Brazil. PLoS One. 2016;11:e0145954. 10.1371/journal.pone.0145954. 10.1371/journal.pone.0145954 PMC4706314 26745799

[21] Anjum SI, Ullah A, Khan KA, Attaullah M, Khan H, Ali H, Bashir MA, Tahir M, Ansari MJ, Ghramh HA, Adgaba N, Dash CK (2019). Composition and functional properties of propolis (bee glue): a review. Saudi J Biol Sci.

[22] Park YK, Alencar SM, Aguiar CL (2002). Botanical origin and chemical composition of Brazilian Propolis. J Agric Food Chem.

[23] Daugsch A, Moraes CS, Fort P, Park YK (2008). Brazilian red Propolis - chemical composition and botanical origin. Evidence-Based Complement Altern Med.

[24] Ferreira JM, Fernandes-Silva CC, Salatino A, Negri G, Message D (2017). New propolis type from north-East Brazil: chemical composition, antioxidant activity and botanical origin. J Sci Food Agric.

[25] Devequi-Nunes D, Machado BAS, Barreto G de A, Silva JR, Silva DF da, Rocha JLC da, Brandão HN, Borges VM, Umsza-Guez MA. Chemical characterization and biological activity of six different extracts of propolis through conventional methods and supercritical extraction. PLoS One. 2018;13:e0207676. 10.1371/JOURNAL.PONE.0207676. 10.1371/journal.pone.0207676 PMC6279037 30513100

[26] Machado BAS, Barreto G de A, Costa AS, Costa SS, Silva RPD, da Silva DF, Brandão HN, Rocha JLC, Nunes SB, Umsza-Guez MA, Padilha FF (2015) Determination of parameters for the supercritical extraction of antioxidant compounds from green Propolis using carbon dioxide and ethanol as co-solvent. PLoS One 10:e0134489. 10.1371/journal.pone.0134489. 10.1371/journal.pone.0134489 PMC4529176 26252491

[27] Chemat F, Rombaut N, Sicaire A-G, Meullemiestre A, Fabiano-Tixier A-S, Abert-Vian M (2017). Ultrasound assisted extraction of food and natural products. Mechanisms, techniques, combinations, protocols and applications. A review. Ultrason Sonochem.

[28] Both S, Chemat F, Strube J (2014). Extraction of polyphenols from black tea – conventional and ultrasound assisted extraction. Ultrason Sonochem.

[29] ANVISA - Agência Nacional de Vigilância Sanitária. Detecção e Identificação dos Fungos de Importância Médica 2004. https://www.anvisa.gov.br/servicosaude/microbiologia/mod_7_2004.pdf. Accessed 7 Aug 2021.

[30] Madhavan P, Jamal F, Chong PP, Ng KP. Identification of local clinical Candida isolates using CHROMagar Candida TM as a primary identification method for various Candida species. Trop Biomed. 2011;28:269–74. PMID: 22041745. 22041745

[31] White T, Bruns T, Lee S, Taylor J, Innis M, Gelfand D, Sninsky J, White T (1990). Amplification and direct sequencing of fungal ribosomal RNA genes for phylogenetics. PCR protocols: a guide to methods and applications.

[32] Reis JH de O, Barreto G de A, Cerqueira JC, Anjos JP dos, Andrade LN, Padilha FF, Druzian JI, Machado BAS. Evaluation of the antioxidant profile and cytotoxic activity of red propolis extracts from different regions of northeastern Brazil obtained by conventional and ultrasound-assisted extraction. PLoS One. 2019;14:e0219063. 10.1371/journal.pone.0219063. 10.1371/journal.pone.0219063 PMC6611595 31276476

[33] Clinical and Laboratory Standards Institute (CLSI). M27-S3: Reference Method for Broth Dilution Antifungal Susceptibility Testing of Yeasts; Third Informational Supplement. 3 rd. Wayne, Pennsylvania; 2008. http://medicine.kaums.ac.ir/UploadedFiles/angalshenase/M27-S3ThirdInternational Supplement.pdf.

[34] Rodríguez-Tudela JL, Berenguer J, Martínez-Suárez JV, Sanchez R (1996). Comparison of a spectrophotometric microdilution method with RPMI-2% glucose with the National Committee for clinical laboratory standards reference macrodilution method M27-P for in vitro susceptibility testing of amphotericin B, flucytosine, and flucona. Antimicrob Agents Chemother.

[35] Clinical and Laboratory Standards Institute (CLSI). M27-S4 - Reference Method for Broth Dilution Antifungal Susceptibility Testing of Yeasts; Fourth Informational Supplement. Wayne, Pennsylvania; 2012. https://webstore.ansi.org/standards/clsi/clsim27s4.

[36] Lohse MB, Gulati M, Valle Arevalo A, Fishburn A, Johnson AD, Nobile CJ (2017). Assessment and optimizations of Candida albicans in vitro biofilm assays. Antimicrob Agents Chemother.

[37] Kalil MA, Santos LM, Barral TD, Rodrigues DM, Pereira NP, Sá M da CA, Umsa-Guez MA, Machado BAS, Meyer R, Portela RW. Brazilian green Propolis as a therapeutic agent for the post-surgical treatment of Caseous lymphadenitis in sheep. Front Vet Sci. 2019;6:399. 10.3389/fvets.2019.00399. 10.3389/fvets.2019.00399 PMC6887654 31850377

[38] Gendreau L, Loewy ZG (2011). Epidemiology and etiology of denture stomatitis. J Prosthodont.

[39] Gauch LMR, Pedrosa SS, Silveira-Gomes F, Esteves RA, Marques-da-Silva SH (2018). Isolation of Candida spp. from denture-related stomatitis in Pará, Brazil. Brazilian J Microbiol.

[40] Mahdavi Omran S, Rezaei Dastjerdi M, Zuashkiani M, Moqarabzadeh V, Taghizadeh-Armaki M (2018). In vitro antifungal susceptibility of Candida species isolated from Iranian patients with denture stomatitis. Biomed Res Int.

[41] Miranda-Cadena K, Marcos-Arias C, Mateo E, Aguirre JM, Quindós G, Eraso E (2018). Prevalence and antifungal susceptibility profiles of Candida glabrata, Candida parapsilosis and their close-related species in oral candidiasis. Arch Oral Biol.

[42] Neppelenbroek K, Seó R, Urban V, Silva S, Dovigo L, Jorge JH, Campanha NH (2014). Identification of Candida species in the clinical laboratory: a review of conventional, commercial, and molecular techniques. Oral Dis.

[43] Martins KV, Gontijo SM de L. Tratamento da estomatite protética: revisão de literatura. Rev Bras Odontol. 2017;74:215. 10.18363/rbo.v74n3.p.215.

[44] Pereira GH, Müller PR, Szeszs MW, Levin AS, Melhem MSC (2010). Five-year evaluation of bloodstream yeast infections in a tertiary hospital: the predominance of non- C. albicans Candida species. Med Mycol.

[45] dos Santos ER, Forno CFD, Hernandez MG, Kubiça TF, Venturini TP, Chassot F, Santurio JM, Alves SH (2014). Susceptibility of Candida spp. isolated from blood cultures as evaluated using the M27-A3 and the new M27-S4 aproved breakpoints. Rev Inst Med Trop Sao Paulo.

[46] Mahmoudabadi AZ, Najafyan M, Alidadi M (2010) Clinical study of Candida vaginitis in Ahvaz, Iran and susceptibility of agents to topical antifungal. Pak J Med Sci 26:607–610. www.pjms.com.pk607. Accessed 7 Aug 2021.

[47] Sadeghi G, Ebrahimi-Rad M, Mousavi SF, Shams-Ghahfarokhi M, Razzaghi-Abyaneh M (2018). Emergence of non- Candida albicans species: epidemiology, phylogeny and fluconazole susceptibility profile. J Mycol Med.

[48] Schorling SR, Kortinga HC, Froschb M, Mühlschlegel FA (2000). The role of Candida dubliniensis in Oral candidiasis in human immunodeficiency virus-infected individuals. Crit Rev Microbiol.

[49] Aslani N, Janbabaei G, Abastabar M, Meis JF, Babaeian M, Khodavaisy S, Boekhout T, Badali H (2018). Identification of uncommon oral yeasts from cancer patients by MALDI-TOF mass spectrometry. BMC Infect Dis.

[50] Moran GP, Sullivan DJ, Henman MC, McCreary CE, Harrington BJ, Shanley DB, Coleman DC (1997). Antifungal drug susceptibilities of oral Candida dubliniensis isolates from human immunodeficiency virus (HIV)-infected and non-HIV-infected subjects and generation of stable fluconazole-resistant derivatives in vitro. Antimicrob Agents Chemother.

[51] Ruhnke M (2000). Development of simultaneous resistance to fluconazole in Candida albicans and Candida dubliniensis in a patient with AIDS. J Antimicrob Chemother.

[52] Ernst EJ, Klepser ME, Pfaller MA (2000). Postantifungal effects of Echinocandin, azole, and Polyene antifungal agents against Candida albicans and Cryptococcus neoformans. Antimicrob Agents Chemother.

[53] Hazirolan G, Canton E, Sahin S, Arikan-Akdagli S (2013). Head-to-head comparison of inhibitory and fungicidal activities of fluconazole, Itraconazole, Voriconazole, Posaconazole, and Isavuconazole against clinical isolates of Trichosporon asahii. Antimicrob Agents Chemother.

[54] Krishnasamy L, Krishnakumar S, Kumaramanickavel G, Saikumar C (2018). Molecular mechanisms of antifungal drug resistance in Candida species. J Clin Diagnostic Res.

[55] Lemos J de A, Costa CR, Araújo CR de, Souza LKH e, Silva M do RR. Susceptibility testing of Candida albicans isolated from oropharyngeal mucosa of HIV+ patients to fluconazole, amphotericin B and caspofungin: killing kinetics of caspofungin and amphotericin B against fluconazole resistant and susceptible isolates. Brazilian J Microbiol. 2009;40:163–9. 10.1590/S1517-83822009000100028. 10.1590/S1517-838220090001000028 PMC3768489 24031337

[56] Jorge R, Furtado NAJC, Sousa JPB, da Silva Filho AA, Gregório Junior LE, Martins CHG (2008). Brazilian Propolis: seasonal variation of the Prenylated p -Coumaric acids and antimicrobial activity. Pharm Biol.

[57] Xuan H, Li Z, Yan H, Sang Q, Wang K, He Q, Wang Y, Hu F (2014). Antitumor activity of Chinese Propolis in human breast Cancer MCF-7 and MDA-MB-231 cells. Evidence-Based Complement Altern Med.

[58] Pazin WM, Mônaco L da M, Egea Soares AE, Miguel FG, Berretta AA, Ito AS. Antioxidant activities of three stingless bee propolis and green propolis types. J Apic Res. 2017;56:40–9. 10.1080/00218839.2016.1263496.

[59] Machado JL, Assunção AKM, da Silva MCP, Dos Reis AS, Costa GC, Arruda DDS, Rocha BA, Vaz MMOLL, Paes AMA, Guerra RNM, Berretta AA, Nascimento FRF (2012). Brazilian green Propolis: anti-inflammatory property by an Immunomodulatory activity. Evidence-Based Complement Altern Med.

[60] Messerli SM, Ahn M-R, Kunimasa K, Yanagihara M, Tatefuji T, Hashimoto K, Mautner V, Uto Y, Kumazawa S, Kaji K, Ohta T, Maruta H (2009). Artepillin C (ARC) in Brazilian green propolis selectively blocks oncogenic PAK1 signaling and suppresses the growth of NF tumors in mice. Phyther Res.

[61] Paulino N, Abreu SRL, Uto Y, Koyama D, Nagasawa H, Hori H, Dirsch VM, Vollmar AM, Scremin A, Bretz WA (2008). Anti-inflammatory effects of a bioavailable compound, Artepillin C, in Brazilian propolis. Eur J Pharmacol.

[62] Yoshimasu Y, Ikeda T, Sakai N, Yagi A, Hirayama S, Morinaga Y, Furukawa S, Nakao R (2018). Rapid bactericidal action of Propolis against Porphyromonas gingivalis. J Dent Res.

[63] López BG-C, Schmidt EM, Eberlin MN, Sawaya ACHF (2014). Phytochemical markers of different types of red propolis. Food Chem.

[64] Dantas Silva RP, Machado BAS, Barreto G de A, Costa SS, Andrade LN, Amaral RG, Carvalho AA, Padilha FF, Barbosa JDV, Umsza-guez MA. Antioxidant, antimicrobial, antiparasitic, and cytotoxic properties of various Brazilian propolis extracts. PLoS One. 2017;12:e0172585. 10.1371/journal.pone.0172585. 10.1371/journal.pone.0172585 PMC5373518 28358806

[65] Lima Cavendish R, de Souza SJ, Belo Neto R, Oliveira Paixão A, Valéria Oliveira J, Divino de Araujo E, Silva AAB, Thomazzi SM, Cardoso JC, Gomes MZ (2015). Antinociceptive and anti-inflammatory effects of Brazilian red propolis extract and formononetin in rodents. J Ethnopharmacol.

[66] Singla RK, Dubey AK (2019). Molecules and metabolites from natural products as inhibitors of biofilm in Candida spp. pathogens. Curr Top Med Chem.

[67] De-Carli AD, Zárate–Pereira P, De-Carli G, Zafalon EJ, Zárate CB de R, Yassumoto LM. Ação da própolis de apis mellifera associada ao fluoreto de sódio sobre o biofilme dental: ensaio clínico duplo cego randomizado. Rev Odontológica do Bras Cent. 2010;19:51. 10.36065/ROBRAC.V19I51.523.

[68] Koo H, Gomes BPFA, Rosalen PL, Ambrosano GMB, Park YK, Cury JÁ (2000). In vitro antimicrobial activity of propolis and Arnica Montana against oral pathogens. Arch Oral Biol.

[69] das Neves MVM, da Silva TMS, de Oliveira Lima E, da Cunha EVL, Oliveira E de J (2016) Isoflavone formononetin from red propolis acts as a fungicide against Candida sp. Brazilian J Microbiol 47:159–166. 10.1016/j.bjm.2015.11.009 PMC4822756 26887239

[70] Machado CS, Mokochinski JB, Lira TO De, de Oliveira F de CE, Cardoso MV, Ferreira RG, Sawaya ACHF, Ferreira AG, Pessoa C, Cuesta-Rubio O, Monteiro MC, Campos MS, Torres YR. Comparative study of chemical composition and biological activity of yellow, green, Brown, and red Brazilian Propolis. Evidence-Based Complement Altern Med. 2016;2016:1–11. 10.1155/2016/6057650. 10.1155/2016/6057650 PMC4972909 27525023

[71] Pippi B, Lana AJD, Moraes RC, Güez CM, Machado M, de Oliveira LFS, von Poser GL, Fuentefria AM (2015). In vitro evaluation of the acquisition of resistance, antifungal activity and synergism of Brazilian red propolis with antifungal drugs on Candida spp. J Appl Microbiol.

[72] Tobaldini-Valerio FK, Bonfim-Mendonça PS, Rosseto HC, Bruschi ML, Henriques M, Negri M, Silva S, Svidzinski TI (2016). Propolis: a potential natural product to fight Candida species infections. Future Microbiol.

[73] Bezerra CRF, Assunção Borges KR, Alves R de NS, Teles AM, Pimentel Rodrigues IV, da Silva MACN, Nascimento MDDSB, Bezerra GFB. Highly efficient antibiofilm and antifungal activity of green propolis against Candida species in dentistry materials. PLoS One. 2020;15:e0228828. 10.1371/journal.pone.0228828. 10.1371/journal.pone.0228828 PMC7757894 33362254

[74] Capoci IRG, Bonfim-Mendonça PDS, Arita GS, Pereira RRDA, Consolaro MEL, Bruschi ML, Negri M, Svidzinski TI (2015). Propolis is an efficient fungicide and inhibitor of biofilm production by vaginal Candida albicans. Evidence-Based Complement Altern Med.

[75] Nani BD, Sardi J de CO, Lazarini JG, Silva DR, Massariolli AP, Cunha TM, de Alencar SM, Franchin M, Rosalen PL. Anti-inflammatory and anti- Candida effects of Brazilian organic Propolis, a promising source of bioactive molecules and functional food. J Agric Food Chem. 2020;68:2861–71. 10.1021/acs.jafc.8b07304. 10.1021/acs.jafc.8b07304 31369255

[76] CDC. Antibiotic Resistance Threats in the United States. Atlanta; 2019. 10.15620/cdc:82532.

