# Supplementary material for: Activity of antifungal drugs and Brazilian red and green propolis extracted with different methodologies against oral isolates of Candida spp
Source: BMC Complement Med Ther. 2021 Nov 24;21:286. doi: 10.1186/s12906-021-03445-5 (PMC8611924; doi:10.1186/s12906-021-03445-5)
Supplement: Supplementary file 1 — Additional file 1. [file 12906_2021_3445_MOESM1_ESM.doc]

**Table S1 - Determination of the chemical composition and antioxidant activity of the green and red propolis extracts used in this study.** The quantification of p-coumaric acid, artepilin C, formononetin and kaempferol was performed using a high-performance liquid chromatography (HPLC) system equipped with an automatic injector and diode array detector (DAD). The content of phenolic compounds was achieved based on the reaction with the Folin-Ciocalteau reagent, followed by spectrophotometry analysis at 765 nm. The content of total flavonoid compounds was determined using a method based on the reaction with a 2% methanol solution of aluminum chloride, followed by spectrophotometry analysis at 415 nm. The antioxidant activity was assessed using the 1,1-diphenyl2-picrilidrazil (DPPH) method. GP_EtOH: green propolis ethanolic extract without ultrasound pretreatment; GP_US: green propolis ethanolic extract with ultrasound pretreatment; RP EtOH: red propolis ethanolic extracts without ultrasound pretreatment and RP_US: red propolis ethanolic extract with ultrasound pretreatment.

| **Compound/Activity** | **GP_EtOH^a^** | **GP_US^b^** | **RP_EtOH^c^** | **RP_US^c^** |
| --- | --- | --- | --- | --- |
| Phenolic Compounds (mg EAG/g) | 181.71±0.01 | 342.09±0.08 | 308.49±6.91 | 314.49±14.00 |
| Flavonoids (mg EQ/g) | 46.80±0.01 | 22.68±0.06 | 82.42±4.45 | 90.38±3.36 |
| p-coumaric acid (µg/mL) | 24.65±0.24 | 8.1±0.007 | n.d. | n.d. |
| Artepilin C (µg/mL) | 569.85±0.11 | 506.77±0.12 | n.d. | n.d. |
| Formononetin (mg/g) | 4.54±0.01 | 7.77±0.02 | 8.68±0.01 | 8.40±0.01 |
| Kaempferol (mg/g) | 1,054±0.01 | 0.913±0.001 | 0.88±0.00 | 0.51±0.00 |
| Antioxidant Activity by DPPH (IC_50_) | 31.80±0.16 | 106.20±12.51 | 76.58±4.17 | 72.70±3.01 |

ᵃ Reference [20]

ᵇ Unpublished results

ᶜ Reference [32]

n.d.= not determined (according to the authors, the concentration of these compounds in these extracts were below the minimum limits of detection)
